# Interlayer Bond Strength Testing in 3D-Printed Mineral Materials for Construction Applications

**DOI:** 10.3390/ma15124112

**Published:** 2022-06-09

**Authors:** Izabela Hager, Marcin Maroszek, Katarzyna Mróz, Rafał Kęsek, Marek Hebda, Leonid Dvorkin, Vitaliy Marchuk

**Affiliations:** 1 Chair of Building Materials Engineering, Faculty of Civil Engineering, Cracow University of Technology, 24 Warszawska Street, 31-155 Krakow, Poland; katarzyna.mroz@pk.edu.pl (K.M.); kesekrafal@gmail.com (R.K.); 2 Doctoral School, Cracow University of Technology, 24 Warszawska Street, 31-155 Krakow, Poland; marcin.maroszek@doktorant.pk.edu.pl; 3 Chair of Materials Engineering, Faculty of Materials Engineering and Physics, Cracow University of Technology, 37 Jana Pawła II Street, 31-864 Krakow, Poland; marek.hebda@pk.edu.pl; 4 Department of Building Elements Technology and Materials Science, National University of Water and Environmental Engineering, 33028 Rivne, Ukraine; l.i.dvorkin@nuwm.edu.ua (L.D.); v.v.marchuk@nuwm.edu.ua (V.M.)

**Keywords:** additive manufacturing, building materials, 3D printing, interlayer bond strength

## Abstract

There are no standards for testing the properties of 3D-printed materials; hence, the need to develop guidelines for implementing this type of experiment is necessary. The work concerns the development of a research methodology for interlayer bond strength evaluation in 3D-printed mineral materials. In additive manufactured construction elements, the bond strength is a significant factor as it determines the load-bearing capacity of the entire structural element. After we completed a literature review, the following three test methods were selected for consideration: direct tensile, splitting, and shear tests. The paper compares the testing procedure, results, and sample failure modes. The splitting test was found to be the most effective for assessing layer adhesion, by giving the lowest scatter of results while being an easy test to carry out.

## 1. Introduction

Three-dimensional (3D) printing technology, from its first use in the 1980s, has gradually started to play an increasingly important role in all industries, especially technical ones. Consequently, 3D printing has also found wide application in the construction industry, becoming a fully-fledged production process. The growing development of the global construction industry poses more and more challenges, which are the driving force for the development of 3D-printing technology. The 3D-printing technologies, compared with traditional techniques of constructing buildings, could be considered an energy-saving and environmentally friendly derivative, giving almost unlimited possibilities for geometric complexity realizations. Numerous advantages of this technology, such as reducing the costs and time of manufacturing [1], minimizing the pollution of the environment, and decreasing the waste produced on the building site, have enhanced its popularity and interest recently. It also creates the possibility of building structures that are impossible or too expensive to develop with the use of traditional construction techniques [2]. In addition, a significant amount of waste that is generated in the conventional construction process, especially from the formwork, is not produced when using the 3D-printing method.

The use of 3D-printing technology presents many challenges. The fundamental issue is ensuring the correct selection of the mix, which will exhibit appropriate rheological properties in the fresh state, but also ensure satisfactory mechanical performances when hardened. The use of setting and hardening accelerators (addition of activators, i.e., Ca(OH)_2_) facilitates the 3D-printing process. Sometimes retardants are needed, and saccharose is used to regulate the setting time [3].

The designing process of mixes is also quite a challenging task requiring mainly an experimental approach. The key parameters that need to be tested to verify the applicability of the concrete mix to the 3D-printing method are workability, pumpability, open-time, and extrudability. Moreover, the material for 3D printing must have adequate fluidity to facilitate extrusion through the nozzle.

A material has to be designed to carry the load of the successive layers. The 3D-printed element created with a material characterized by a high fluidity is highly prone to buckling and collapse. In 3D-printing technology, rheology and adequate fluidity are key factors for successful printing [4,5]. If the mix is too stiff, it could weaken the bond strength because the mixture is too dry.

**Figure 1:**
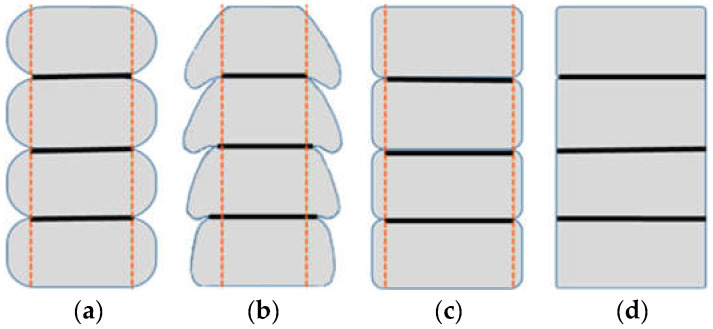
The geometry of printouts and the optimum interlayer bond: (**a**) with round edge, (**b**) with large coves, (**c**) reduced curvature of edge, (**d**) rectangular cross-section.

Depositing a layer on a layer determines the formation of connections between them, which are the weakest link in the entire structure [6]. To increase the strength of the interlayer bonds, appropriate geometry should be ensured. A rectangular cross-section of the printed path with a maximum bonding surface is desirable (Figure 1d). In the case of large coves, the material may detach from the layer above (Figure 1b). Reducing the curvature results in a larger area of force transfer to the lower layer (Figure 1c,d), which can be obtained when levelling trowels are used.

The interlayer bond strength depends on the time it takes for the print head to pass. Choosing the right time interval between the application of subsequent layers is crucial and identifying the maximum transition time of the head gives the appropriate connection between the layers. The adequate gap time will guarantee the avoidance of the so-called “cold joint” [7] with low strength parameters. To improve the quality of the bond between the layers, an appropriate selection of the time interval between the application of subsequent layers needs to be selected [8]. A longer time interval causes the formation of voids and cavities between the layers. Empty spaces dominate the structures where the time gap increases. It is caused by the loss of surface moisture (cold joint) along with the extension of the time interval, which results in a decrease in the bond strength.

Research on the influence of the surface roughness by different surface modification techniques (wire brushing, addition of sand or cement, and moisturizing substrate layer) is presented in [8]. The substrate surface roughness was measured and the interlayer bond strength was investigated, showing the strength development when combing was applied.

The nozzle distance also has a significant influence on the interlayer bond strength. Weaker inter-layer bonding forces were achieved for a greater distance between the nozzle and the printed structure [9]. A reduction in bond strength occurred by about 23% for the offset of 2 mm and by about 35% for the offset of 4 mm [10]. As the nozzle distance increased, more voids were observed between the layers, which harmed the bond strength [10].

## 2. Mechanical Properties Testing of 3D-Printed Materials

The hardened 3D printout is considered an orthotropic material because of the layered structure of printouts [11]. As a consequence of the production process, the material properties depend on the direction of observation. The orthotropy of 3D prints made with the use of additive technologies is caused by the layered structure of the printout. It is necessary to ensure an appropriate connection between the layers so as to ensure structural integrity. It should be emphasized that still there are no standards for testing the properties of printed materials; hence, the development of guidelines or recommendations for the implementation of this type of experiment is highly needed.

**Figure 2:**
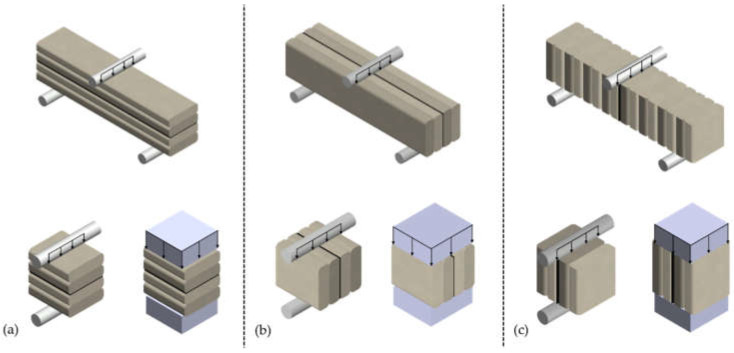
Schemes of strength test configurations following the direction of printing: (**a**) perpendicular to the layer–interface plane, (**b**) cross-section, (**c**) side.

The orthotropy of the properties of printed materials is considered to be one of the main disadvantages of structures printed with using additive method [9]. This determines the need to test the prints in terms of their mechanical properties. Scientists adopt different types of experiments. Usually, strength tests are configured following the direction of printing, i.e., perpendicular, cross-section, and side (Figure 2).

**Figure 3:**
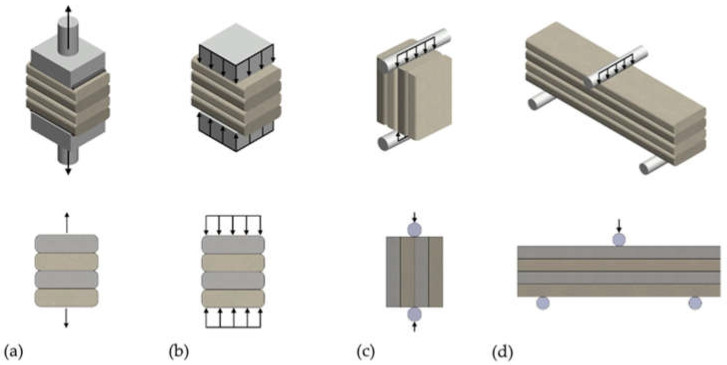
Schemes of strength test for 3D prints: (**a**) direct tensile test, (**b**) compressive strength test, (**c**) splitting test, and (**d**) three-point bending test.

The most common strength tests carried out on 3D prints are the (a) direct tensile test, (b) compressive strength test, (d) splitting test, and (c) three-point bending test, as presented in Figure 3.

Sometimes, samples are prepared for testing by cutting off the side notches. In some studies, specimens are trimmed to cut the overhangs pressed out of the layers, as shown in Figure 1. Such sample trimming does not need to be used when layer levelling trowels are used. It has been reported that trimming samples improves the homogeneity of the results because of the even and easily determinable surface area of the loaded surface [12].

## 3. Bond Strength Evaluation

The interlayer bond strength is one of the key aspects of successful 3D-printing technology. Testing of the interlayer bond strength requires choosing an appropriate method to evaluate the bond strength between layers and understanding the bond behavior. There are no standardized methods for determining the inter-layer bond strength in 3D printing, and the research so far is based on the methods of measuring bond strength, inter alia, for concrete or wood [13].

**Figure 4:**
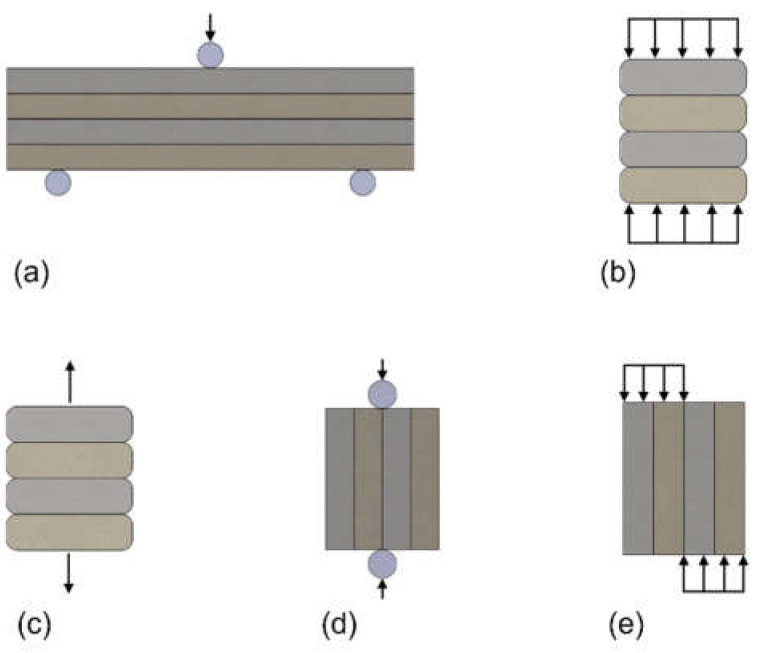
Interlayer bond test methods: (**a**) bending test, (**b**) compression test, (**c**) tensile test, (**d**) splitting tensile test, and (**e**) shear test.

The methods used to measure the interlayer bond strength are direct tension test, splitting test, compressive strength test, bending test, and shear test, as presented in Figure 4.

Scientists’ views on compression tests as a method of interlayer bond strength evaluation are divided. Zhang et al. [14] conducted a compression test on samples cut from the printed element. The compression test was carried out on three axes: X, Y, and Z [14]. The highest compressive strength values were obtained in the case where the layers of material were arranged in a direction parallel to the load. Wolf [7] and Sanjan [15] considered the compression test in the direction of printing as the inter-layer bonding test. According to Nerella [9], the application of the compression test to determine the interlayer bond strength is a questionable issue.

The lack of a standardized method or recommendation on this issue illustrates the need to define a reliable testing method to evaluate the quality of adhesions between the 3D-printed material layers.

The above-mentioned methods for measuring the interlayer bond strength of 3D prints are poorly described in the literature. No comparison study showing differences between the measured bond strength was found in the literature. This is probably the result of the relatively short time that 3D-printing technology has been in use, especially in the construction industry. To overcome these shortcomings, the present research was carried out with the use of the three methods of interlayer bond evaluation: direct tensile test, splitting test, and shear test.

The aim of the work was the development of a stand for testing the bond strength of the layers in mineral-printed materials. The conducted review of the test methods to determine the bond between the layers indicated several possible approaches. Three were chosen to determine the interlayer bond strength: method A, the direct tensile strength test; method B, the splitting test; and method C, the shear strength test.

## 4. Materials

**Figure 5:**
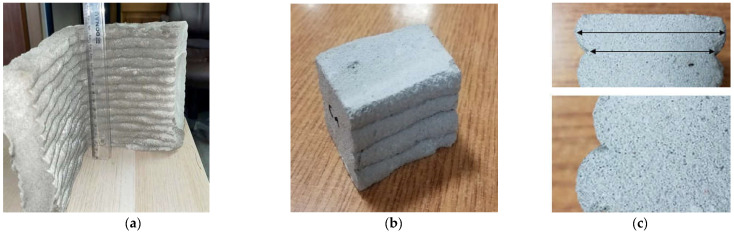
3D-printed element (**a**) before cutting into samples; (**b**) four layers samples 40 × 50 × 45 mm; (**c**) cross-section of the contact zone between the layers.

The test samples were taken from a commercially 3D-printed element, as seen in Figure 5. The composition of the cement-based composite and the 3D-printing procedure have been reserved by the manufacturer. The printed element was cut into four-layer samples with cross-section dimensions of 40 mm × 50 mm, as seen in Figure 5a. For this purpose, a table tile saw was used to cut the rocks using the wet method. The cutting process was carried out 30 days after the production of the printed elements. The loaded surfaces of the samples were additionally levelled and polished on a grinding stone to ensure smoothness. The size of the samples was selected to ensure sample representativeness, and we adjusted the size to enable the measurements (four layers), as seen in Figure 5b. Figure 5c shows the appearance of the contact zone between the layers. A good connection between the layers was achieved because there were no air bubbles or a clear dividing line between the layers. The structure of the material was homogenous with visually evenly distributed pores.

## 5. Material Strength and Bond Strength Testing

A ZwickRoell 50 kN testing machine was used to perform the tests. This is a flexible testing machine that can carry out compression, direct tensile, shear, and splitting tests. The loading was adopted in the preliminary tests.

### 5.1. Compressive Strength

**Figure 6:**
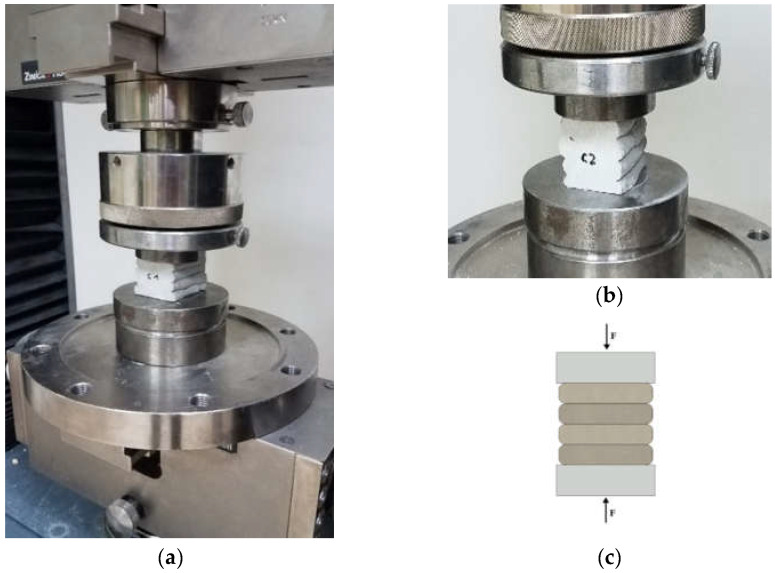
Compression test of 3D-printed materials: (**a**) testing stand for a compression test, (**b**) sample on the test bench under loading, and (**c**) the test scheme.

Compressive strength was tested on six samples. The samples were labelled from C1 to C6, respectively. The load was transferred to the sample at a speed of 500 N/s until failure. The head of the testing machine was assembled with a joint to adapt to any possible non-parallelism of the surface. In Figure 6, the testing stand is presented with the sample configuration for compressive strength.

The following Formula (1) was used to calculate the compressive strength for the surface area, and the minimum cross-section was determined for the narrowest cross-section.

(1) $$\sigma_{max=}\frac{F_{max}}{S}$$

where

$\sigma_{max}$ is the maximum stress in compression, compressive strength (MPa);
$F_{max}$ is the load at failure (kN);
S is the minimum cross-section determined for the narrowest cross-section (${\mathrm{mm}}^{2}$).

### 5.2. Direct Tensile Strength–Interlayer Bond Strength Test: Method 

**Figure 7:**
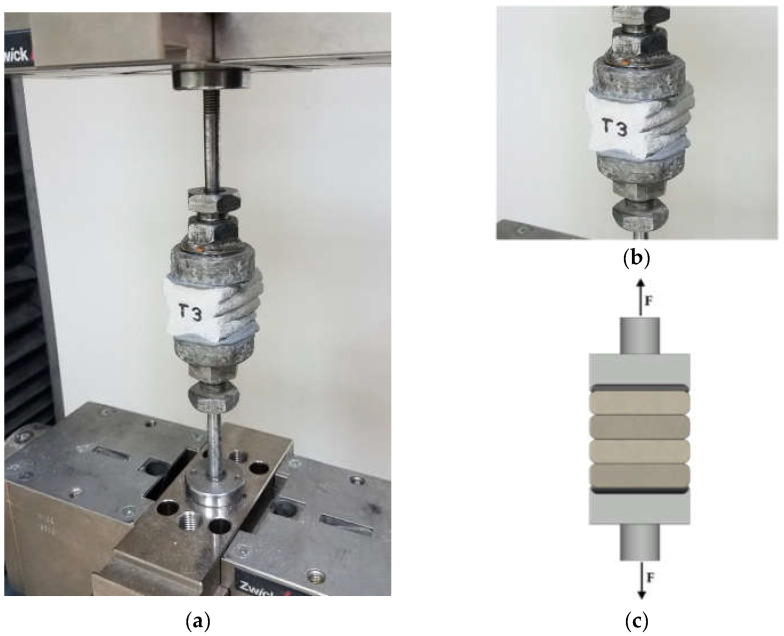
Direct tensile strength–interlayer bond strength test, method A: (**a**) testing stand for direct tensile test, (**b**) sample with steel casts, and (**c**) the test scheme.

The tensile strength test was carried out on samples cut in the same way as those for the compressive strength test. The direct tensile method was adopted to determine the interlayer bond strength. On the surface of the samples with dimensions of about 40 × 50 × 45 mm, steel casts with a diameter of 5 cm were glued using the two-component adhesive. The samples and the steel casts used are shown in Figure 7a. The sample was placed in a testing machine, then the tensile test was performed according to the scheme presented in Figure 7b,c.

The direct tensile strength was performed on three samples. The samples were labelled T1, T2, and T3, respectively. The load was transferred to the sample at a speed of 100 N/s until failure. In the upper loading head, the articulations were used, which enabled even loading of the samples and eliminated undesirable stresses due to non-axial load application. The test methodology allowed for determining the maximum tensile stresses that the material could transfer during a static direct tensile test. At the same time, it allows for determining the adhesion of printed layers using the direct tensile method.

Formula (2) was used to calculate the maximum tensile stresses and to determine the bond strength of the layers. The surface area was determined after failure occurred in the place with the smallest cross-section.

(2) $$\sigma_{maxA=}\frac{F_{max}}{S}$$

where

$\sigma_{maxA}$ is the maximum stress in the direct tension, the tensile strength/interlayer bond strength method A (MPa);
$F_{max}$ is the maximum load (kN);
S is the surface area in the ruptured zone (${\mathrm{mm}}^{2}$).

### 5.3. Splitting Test–Interlayer Bond Strength Test: Method B

The splitting strength test was carried out on three samples. The samples were labelled Z1, Z2, and Z3, respectively. The samples were approximately 40 × 50 × 45 mm. The samples were placed in the machine and loaded with cylindrical steel heads. The load was transferred to the sample at a constant speed of 50 N/s, until failure.

**Figure 8:**
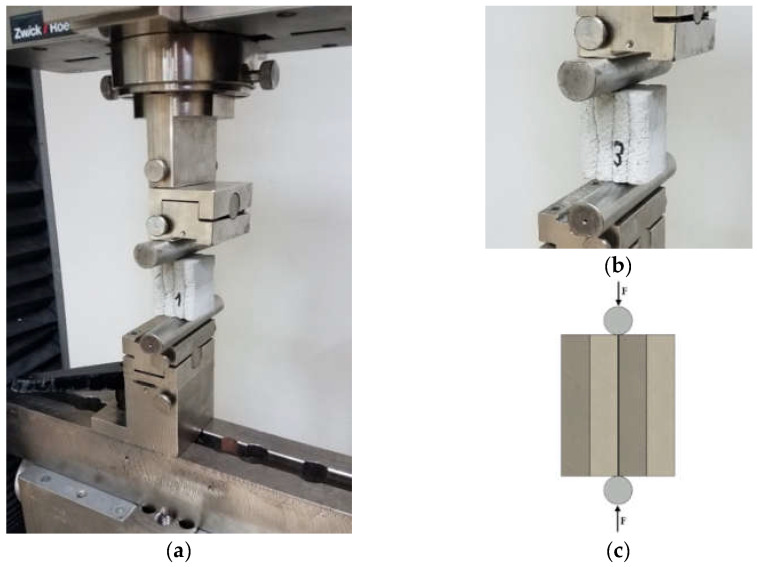
Splitting test–interlayer bond strength test, method B: (**a**) testing setup for splitting test, (**b**) sample during the test, and (**c**) the test scheme.

The test was performed using the setup presented in Figure 8. This method was adopted from the concrete splitting tensile strength test for prisms (Brazilian method) [16]. The following formula was used to calculate the splitting strength (method B):

(3) $$\sigma_{maxB=}\frac{2F_{max}}{\pi dL}$$

where

$\sigma_{max\;B}$ is the maximum stress in splitting, splitting tensile strength/interlayer bond strength method B (MPa);
$F_{max}$ is the maximum load (N);
d is the sample width (mm);
L is the sample length (mm).

### 5.4. Direct Shear Strength Test–Interlayer Bond Strength Test: Method C

**Figure 9:**
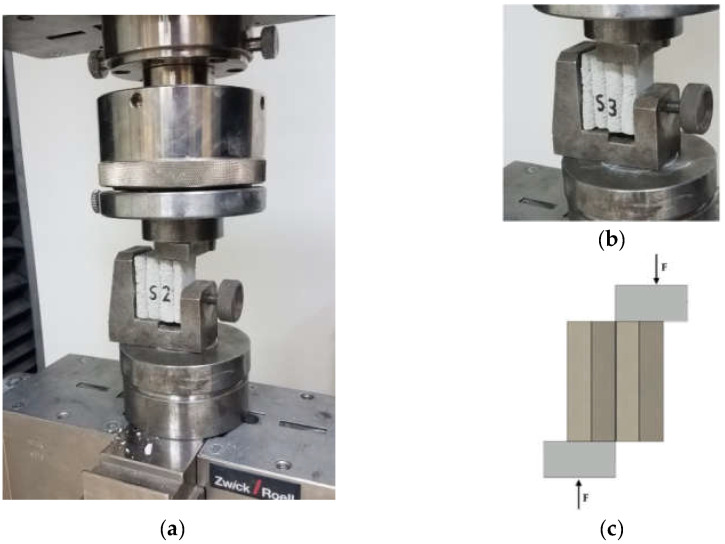
Direct shear strength test–interlayer bond strength test, method C: (**a**) testing stand for shear strength test, (**b**) sample during the test, and (**c**) the test scheme.

The shear strength test was carried out on previously prepared samples that were placed in a steel holder. As in the case of other tests, the samples were cut and the surfaces were polished. Interlayer bond by a shear stresses application test was tested on three samples. The samples were marked S1, S2, and S3, respectively. The load was transferred to the sample at a speed of 50 N/s until failure. Figure 9a shows the test stand during the test. The sample was placed in a steel holder to ensure proper positioning during the test (Figure 9b). The selected test methodology allowed for determining the maximum stresses occurring in shear and to determine the quality of layer adhesion in the printed material.

The following formula (4) was used to calculate the shear strength (method C).

(4) $$\tau_{maxC=}\frac{F_{max}}{S}$$

where

$\tau_{maxC}$ is the maximum stresses when a shear load is applied (MPa);
$F_{max}$ is the maximum load (kN);
*S* is the cross-sectional surface area at the failure zone (${\mathrm{mm}}^{2}$).

## 6. Results

### 6.1. Compressive Strength Test Results

**Figure 10:**
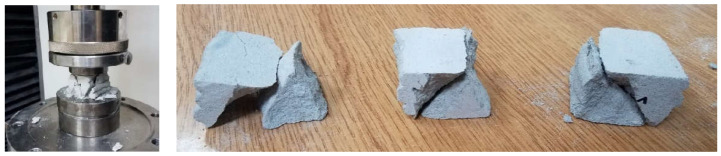
Representative example of samples after the compressive strength test and the mode of rupture of samples, from the right: C1, C2, and C3.

The results obtained during the compressive strength tests are presented in Table 1. The sample failure mode is shown in Figure 10. The compressive strength of the tested 3D-printed material ranged from 16.08 MPa to 18.66 MPa, and the average strength was 17.15 MPa. The obtained results of the compressive strength determined in the transverse direction of the layer system indicated the high homogeneity of the material. The standard deviation was 0.95 MPa with a variance of 0.903 MPa. The failure mode was typical for compression.

**Table 1:** Compressive strength test results.

Sample	*S* (mm^2^)	Force (kN)	Compressive Strength (MPa)
C1	2040.0	32.80	16.08
C2	2025.8	33.50	16.54
C3	2105.3	37.00	17.57
C4	1992.0	32.30	16.21
C5	2009.4	37.50	18.66
C6	2128.0	38.10	17.90

Mean value 17.15 MPa; standard deviation σ = 0.95 MPa, variance, σ^2^ = 0.903 MPa.

### 6.2. Results of Direct Tensile Strength–Interlayer Bond Strength Test: Method A

**Figure 11:**
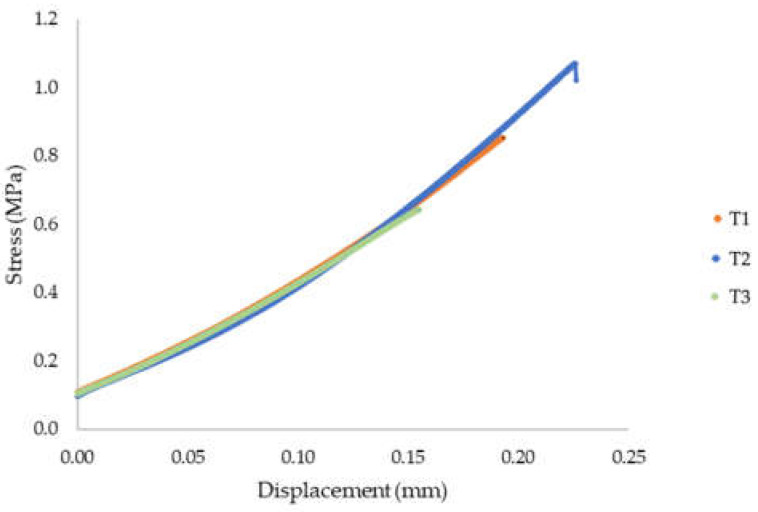
Stress evolution—direct tensile strength/interlayer bond strength (method A).

**Figure 12:**
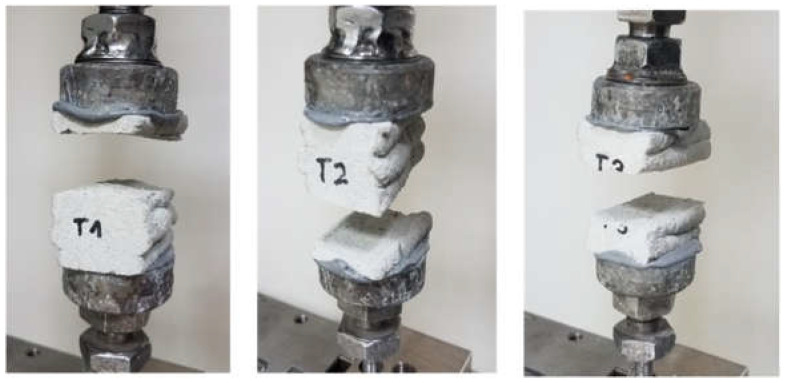
Samples after the direct tensile strength test and the mode of rupture of samples T1, T2, and T3.

Table 2 presents the results of the direct tensile strength and bond strength. Figure 11 presents the evolution of stress as a function of displacement. The sample failure mode is shown in Figure 12. The tensile strength of the tested material for T1 was 0.85 MPa, for T2 was 1.07 MPa, and for T3 was 0.64 MPa. The average strength was 0.85 MPa. The obtained tensile strength results, determined in the direction perpendicular to the layer system, indicated a relatively high heterogeneity of results. The standard deviation was 0.175 MPa. Destruction took place at the connection between the layers where the smallest cross-sectional area of the sample was measured.

**Table 2:** Direct tensile strength test results.

Sample	*S* (mm^2^)	Force (kN)	Direct Tensile Strength, Bond Strength (MPa)
T1	2046.2	1.74	0.85
T2	2107.4	2.26	1.07
T3	2106.0	1.35	0.64

Mean value 0.85; standard deviation σ = 0.175 MPa, variance, σ^2^ = 0.030 MPa.

### 6.3. Results of Splitting Test–Interlayer Bond Strength Test: Method B

**Figure 13:**
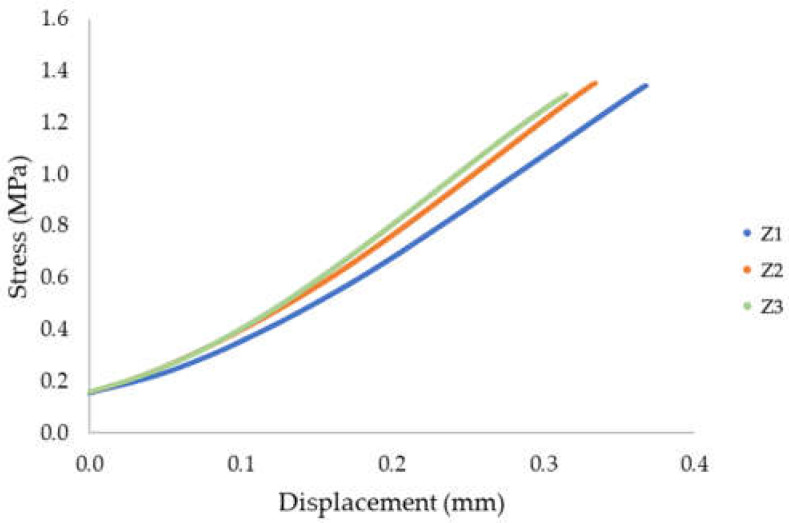
Stress evolution—splitting tensile strength/interlayer bond strength (method B).

**Figure 14:**
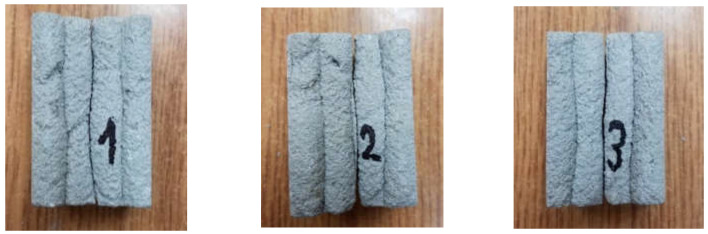
The failure mode of the samples after the splitting test.

The results obtained during the splitting tests (interlayer bond strength) are presented in Table 3. Figure 13 shows a diagram illustrating the evolution of stress as a function of displacement. In Figure 14, the failure mode is presented after the splitting strength test was performed.

**Table 3:** Splitting tensile strength/interlayer bond strength.

Sample	*d* (mm)	*L* (mm)	Force (N)	Splitting Interlayer Bond Strength (MPa)
Z 1	40.8	50.8	4370	1.34
Z 2	40.9	50.9	4400	1.35
Z 3	39.4	49.4	4260	1.34

Mean value 1.34 MPa; standard deviation σ = 0.191 MPa, variance, σ^2^ = 0.036 MPa.

The tensile strength determined by splitting was 1.34 MPa for sample S1, 1.35 MPa for sample S2, and 1.34 MPa for sample S3. The average strength was 1.34 MPa. The obtained results of the splitting tensile strength, determined along the direction of the layer system, were characterized by a high homogeneity. The standard deviation was 0.003 MPa. All of the tested samples were fractured in the joints between the layers, which is visible in Figure 14.

### 6.4. Results of Direct Shear Strength Test–Interlayer Bond Strength Test: Method C

**Figure 15:**
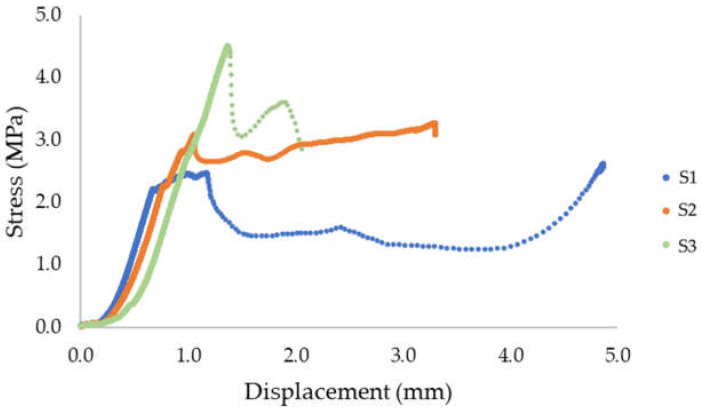
Stress evolution—shear strength/interlayer bond strength (method C).

**Figure 16:**
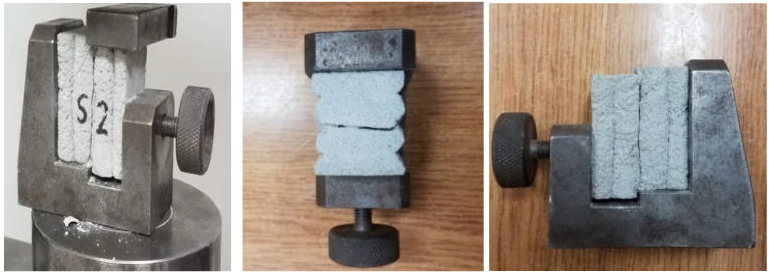
The failure mode of samples after the shear strength test.

The shear strength of the tested material for S1 was 2.62 MPa, S2 was 3.28 MPa, and S3 was 4.51 MPa. The results are presented in Table 4. The average strength was 3.47 MPa. The obtained results of the shear strength show a significant scattering of the results. The standard deviation was 0.78 MPa. Figure 15 shows a diagram that presents the evolution of stress as a function of displacement in the shear strength test. Figure 16 shows the failure mode after the shear strength test was performed. The failure mode of the printed material in the case of samples 1 and 2 did not take place in the place where the layers were joined.

**Table 4:** Shear strength test/interlayer bond strength.

Sample	*S* (mm^2^)	Force (kN)	Shear Strength Bond Strength (MPa)
S1	2062.5	5.40	2.62
S2	2097.1	6.87	3.28
S3	2025.4	9.14	4.51

Mean value 3.47 MPa; standard deviation σ = 0.783 MPa, variance, σ^2^ = 0.613 MPa.

## 7. Conclusions

This paper presents an overview of current knowledge about the mechanical properties testing of 3D-printed mineral materials for construction applications. The main aim of the research concerned bond strength evaluation. Interlayer bond strength is essential when the load-bearing capacity of the 3D-printed element is evaluated because the connection between the layers of printed materials is the “weakest link” in the printed material. Three methods were chosen to determine the interlayer bond strength: method A, direct tensile strength test; method B, splitting test; and method C, shear strength test. The following observations and concussions were drawn:

- The compressive strength of the 3D-printed material evaluated in the direction perpendicular to the layers was 17.15 MPa with a standard deviation of 0.95 MPa.
- The average direct tensile strength (f_t_) determined using the direct tensile method was 0.85 MPa. In the case of this study, failure occurred at the joint point between the layers. Direct tensile strength was also used as method A to determine the interlayer bond strength as the direct method of bond strength evaluation.
- The average layer adhesion determined by the splitting test (method B) was 1.34 MPa. Failure also took place at the inter-layer connection. The method provided the most uniform and reproducible results.
- When testing the strength of the interlayer bond by evaluating shear stresses (method C), the greatest dispersion of results was obtained. The mean value of stresses was 3.47 MPa.
- The direct tensile strength test method requires time-consuming sample preparation, glueing the heads, and cleaning them after testing. The splitting method was found to be the easiest to perform and provided the most homogeneous results (standard deviation σ = 0.191 MPa).
- The greatest repeatability of the obtained results was achieved for the splitting test (method B), as this scheme of testing method forces the failure in the same layers of material compared with the direct method. However, the scatter of results obtained for the shear test highlights this method as being weakly accurate. It is caused by the inaccuracy of the steel support rather than by the inaccuracy of the method. It is expected that after the improvement of support the results will present a similar scatter as for the splitting test.
- The comparison of stresses obtained in the direct method (direct tensile) with indirect methods is unjustified due to the difference in the type of stresses dominating during the test. The way of testing may be adapted to the specific target stresses acting on the material.

It should be emphasized that there are no standards for testing the properties of printed materials; hence, the development of guidelines for implementing this type of experiment is highly needed. However, providing guidelines is a complex achievement and the authors believe that the results presented in the paper may contribute to the enrichment of such a database.

In the authors’ opinion, this test should be based on the splitting testing procedure that was the most effective tool for assessing layer adhesion. The results were repeatable with the lowest result dispersion, and it also observed that the failure mode in this type of test was in the layers joint, which was satisfactory.

It should be noted that the interlayer bond strength depends on the time it takes for the print head to pass. Choosing the right time interval between the application of subsequent layers is crucial, and it needs to guarantee the avoidance of the so-called cold joint with low bond strength parameters. This issue will be investigated in future research.

## Data Availability

Not applicable.
